# Military Tract Medical Association

**Published:** 1868-05

**Authors:** A. H. Thompson, Hiram Nance

**Affiliations:** Sec’y, pro tem.; President


### MILITARY TRACT MEDICAL ASSOCIATION.

The Semi-Annual Meeting of the Military Tract Medical Association was held in Princeton, on Tuesday, Dec. 10, 1867, Dr. Hiram Nance, of Kewanee, in the chair.

The following new members were elected, on report of the Board of Censors: Drs. Kaull, E. Chamberlain, and Dunn, of Bureau Co.; and Prof. Hull, of Iowa Medical College. Dr.

Wm. O. Chamberlin, of Bureau Co., was elected an honorary member.

Dr. S. P. Breed, of Bureau Co., Chairman of Committee on Practical Medicine, read a very interesting report, which, by a vote of the Association, was requested for publication in the CHICAGO MEDICAL EXAMINER.

Dr. Holton, of Bureau Co., presented an interesting report on Materia Medica and Therapeutics, which, on motion, was received.

Dr. Holton offered the following resolution:

*Resolved*, That we, as members of the medical profession, refuse to consult with any physician who as a rule charges but one-half or two-thirds the regular fee, as agreed upon in our fee-bill

After discussion, it was laid upon the table by the casting vote of the President.

Dr. Reece, of Knox Co., presented a very interesting specimen of intestinal concretion, about three inches in length and one and one-third inches in diameter, and supposed to be cholesterine. Dr. R. was appointed a special committee of one, to report fully upon the subject at the next meeting of the Association.

Dr. Reece read an interesting paper on the treatment of fracture of the femur, both simple and compound, without the use of splints, depending upon the use of weights for both extension and counter-extension; also, on the importance and advantages of the silver suture in surgery.

The following Committees were then appointed:

*Surgery*—Drs. A. H. Thompson, Bureau Co.; Madison Reece, Knox Co.; Webster, Warren Co.

*Practical Medicine*—Drs. Holton, Bureau Co.; Boardman, Stark Co.; Breed, Bureau Co.; Brown, Henry Co.

*Materia Medica and Therapeutics*—Drs. Smiley, Henry Co.; Latimer, Bureau Co.; Woodward, Knox Co.

*Obstetrics and Diseases of Women*—Drs. McDill, of Henderson Co.; Phillips, of Knox Co.; Kaull and Crossley, of Bureau Co.

Drs. H. S. Hurd, of Knox Co., and S. P. Breed, of Bureau Co., were appointed special essayists for the next meeting of the Association.

Dr. A. H. Thompson, of Bureau Co., read a paper on organic and functional disease, taking the ground that there could be no functional disease in the strict sense of the term, as all derangements of function must depend on a corresponding derangement of the organism upon which the function depends. On motion, the paper was ordered published.

The Association then adjourned, to meet at Kewanee on the second Tuesday in May next.

